# Why has epidemiology not (yet) succeeded in identifying the origin of the asthma epidemic?

**DOI:** 10.1093/ije/dyad035

**Published:** 2023-04-02

**Authors:** Josep M Antó, Neil Pearce, Jeroen Douwes, Judith Garcia-Aymerich, Lucy Pembrey, Lorenzo Richiardi, Jordi Sunyer

**Affiliations:** ISGlobal, Barcelona Institute for Global Health, Barcelona, Spain; Hospital del Mar Medical Research Institute, Barcelona, Spain; Universitat Pompeu Fabra (UPF), Barcelona, Spain; CIBER Epidemiología y Salud Pública (CIBERESP), Barcelona, Spain; Department of Non-communicable Disease Epidemiology and Medical Statistics, London School of Hygiene and Tropical Medicine, London, UK; Research Centre for Hauora and Health, Massey University, Wellington, New Zealand; ISGlobal, Barcelona Institute for Global Health, Barcelona, Spain; Universitat Pompeu Fabra (UPF), Barcelona, Spain; CIBER Epidemiología y Salud Pública (CIBERESP), Barcelona, Spain; Department of Medical Statistics, London School of Hygiene and Tropical Medicine, London, UK; Cancer Epidemiology Unit, Department of Medical Sciences, University of Turin, and CPO-Piemonte, Torino, Italy; ISGlobal, Barcelona Institute for Global Health, Barcelona, Spain; Hospital del Mar Medical Research Institute, Barcelona, Spain; Universitat Pompeu Fabra (UPF), Barcelona, Spain; CIBER Epidemiología y Salud Pública (CIBERESP), Barcelona, Spain

## Introduction

In the 1980s many industrialized countries experienced large increases in the prevalence of asthma with the term ‘asthma epidemic’ becoming popular.^1^^,^^2^ Since epidemiology had played a leading role in the discovery of the carcinogenic effects of tobacco,^3^ as well as in understanding several risk factors for cardiovascular diseases,^4^ it was hoped that epidemiology would contribute to identifying the causes of the asthma epidemic and to developing adequate prevention strategies. Three decades later, for reasons that have not been sufficiently considered, these expectations have not been fulfilled. Here, we summarize the characteristics of the asthma epidemic, review the most popular attempts at causal explanations and discuss how epidemiology alone may not be sufficient to provide a satisfactory understanding of the asthma epidemic, thus hampering the development of effective interventions.

## The asthma epidemic

The term ‘asthma epidemic’ refers to a secular and geographically widespread increase in the frequency of asthma, which is something different from other types of localized asthma epidemics.^5^^,^^6^ A first defining characteristic of the asthma epidemic is its time trends. In a review, Eder *et al.* showed that the prevalence of asthma, asthma symptoms and atopy had substantially increased from the 1960–70s to the 1990s in many areas of America, Europe and Asia.^7^ Among Finnish conscripts it has been shown that, after a stable period from 1926 to 1961, the prevalence of asthma increased 12-fold between 1966 (0.29%) and 2003 (3.45%), with allergic rhinitis and atopic eczema following a similar trend.^8^ Regarding airway hyper-responsiveness, repeated surveys did not show a consistent secular increase.^7^ Overall, the available evidence indicates a geographically widespread large increase in asthma, allergic rhinitis and atopy prevalence. For eczema, a systematic review found evidence suggesting that the prevalence of atopic eczema was increasing in Africa, Eastern Asia, Western Europe and parts of Northern Europe.^9^

A second characteristic of the asthma epidemic refers to the geographical patterns. The European Community Respiratory Health Survey (ECRHS) studied adults aged 22–44 years in 1990–92 and showed that the prevalence of asthma symptoms varied widely, being generally lower in northern, central and southern Europe and higher in the British Isles, New Zealand, Australia and the USA, with wide variations even within some countries.^10^ The prevalence of nasal allergy,^10^ bronchial responsiveness^11^ and atopy^10^ showed a similar distribution to that of asthma. The International Study on Allergy and Asthma in Children (ISAAC) in the age group of 13–14 years in 1992–93^12^^,^^13^ showed 20- to 60-fold differences between centres in the prevalence of asthma symptoms, allergic rhinoconjunctivitis and atopic eczema. The highest 12-month prevalence rates of asthma were observed in centres in the UK, Australia, New Zealand and the Republic of Ireland; the lowest were in centres in Eastern European countries, Indonesia, Greece, China, Taiwan, Uzbekistan, India and Ethiopia, with an 8-fold variation seen between the 10th and 90th percentiles (3.9–30.6%).^13^ Though the geographical distribution of the prevalence rates of asthma, allergic rhinoconjunctivitis and atopic eczema in ECRHS and ISAAC were similar,^14^ there was a weak to moderate consistency in the geographical distribution of the three phenotypes. Other studies have shown variations in prevalence between centres in proximity^15–17^ and between urban and rural areas in Africa.^18^ As shown by a study in Albania and the UK, large differences in asthma symptoms and exercise-induced bronchial reactivity can occur in populations with similar rates of atopy^19^ suggesting that large geographical variations in asthma prevalence can arise without differences in the frequency of atopy.^7^

A third relevant observation comes from the joint time and geographic differences observed after the epidemic was described. The first follow-up of ECRHS (1998–2003, 10 years after the first survey) reported an increase in nasal allergy as well as in the proportion of participants treated for asthma but not of those reporting symptoms of asthma.^20^^,^^21^ The ISAAC Phase Three study (in 2002–03, 7 years after the first survey)^22^ examined changes in the prevalence of asthma, hay fever and eczema symptoms in a large number of centres worldwide. In the age group of 6–7 years, among the 66 areas included, the prevalence of asthma showed an increase in 25, a decrease in 14 and little change in 27. For the age group of 12–13 years, among 106 areas, the corresponding figures were 42, 40 and 24. Centres showing a decrease were distributed across all continents, though in the age group of 12–13 years they were more likely to be in Western Europe. Patterns of change were similar for allergic rhinoconjunctivitis and eczema. Repeated surveys in Finnish and Russian Karelia suggested that the allergy epidemic occurred only among the Finnish and was mainly attributable to the year of birth.^23^

Considering the evidence, it is possible to suggest that in some Western countries, asthma prevalence began to increase in the 1960s following a heterogenous pattern both in time and geographically that in the 1990s had affected many areas worldwide, but not all, with the areas with the highest prevalence plateauing in the 1990s followed by a decrease. This increase in asthma was paralleled by increases in allergic rhinitis, eczema and atopy, though the increases in asthma prevalence appear to have occurred for both atopic and non-atopic asthma, and probably resulted from both cohort and period effects. The epidemiological observations described above (Table 1) must consistently fit with any causal theory of the asthma epidemic and could be used as a framework for further explanatory attempts.

**Table 1:** The epidemiologic observations that any theory of the asthma epidemic must be able to explain

(i) Asthma prevalence began to increase sometime after the Second World War, i.e. in the 1950s or 1960s
(ii) The changes were very heterogeneous both in time and place, even across centres in proximity
(iii) The increases appear to relate to all of the so-called ‘allergic diseases’, i.e. asthma, allergic rhinitis, allergic conjunctivitis and atopic eczema, but not in a uniform way
(iv) The increases in prevalence appear to have occurred for both atopic and non-atopic asthma
(v) The increases are probably related to both cohort and period factors
(vi) In the countries with the highest prevalence, such as the UK and New Zealand, prevalence appears to have plateaued in the 1990s and may have now begun to decrease

## Causal explanations for the asthma epidemic

### The hygiene hypothesis

In 1985, a hygiene hypothesis was mentioned as an explanation for the appendicitis epidemic in Britain that started at the turn of the twentieth century when infections had begun to decline and sanitation was improving.^24^^,^^25^ In 1989, Strachan reported that hay fever in children increased as the number of siblings decreased and proposed that declines in family size and higher standards of cleanliness could have reduced opportunities for infection and increased atopic disease.^26^ During the 1990s Strachan’s hygiene hypothesis gained wide popularity when the Th1/Th2 paradigm^27^ provided it with some biological plausibility.

The hygiene hypothesis prompted a large number of studies aimed at identifying which type of infection could be associated with a lower risk of asthma, including helminths,^28^ mycobacterial infection and BCG vaccination,^29^ hepatitis A virus,^30^^,^^31^ immunizations against measles and pertussis^32^ and the use of antibiotics.^33^ Although some studies found a protective effect of infections on asthma, others found the opposite or no effect, and there was no consistent evidence that a secular decrease in these infections or the use of antibiotics could have originated the asthma epidemic.

Other studies examined the role of environmental exposures in the hygiene hypothesis, including family size, day care and farming environment. In relation to family size, the negative association between larger family size and lower risk of atopic disease has been consistently replicated,^34^ although the historical change in family size only explains 1% and 5% of the absolute increase in asthma prevalence in England/Wales and New Zealand, respectively.^35^ By contrast, the evidence that attendance at day care could reduce the risk of asthma or allergy is not sufficiently consistent.^36^

Regarding the farming environment, several studies have shown a lower risk of asthma and allergies in people growing up on a farm^37^^,^^38^ and in adult farmers,^39^ with specific protective exposures including contact with animals, consumption of unprocessed cow’s milk and microbial substances in the farm dust.^38^^,^^40^ Studies in humans and mice have shown that exposure to dust in the Amish environment protects against asthma by engaging and shaping the innate immune response.^41^ The protective effect of farming environments has also been reported in farmers in Poland^42^ and Nepal.^43^ The loss of contact with farm environments, which is a common trait in the transition from rural to urban environments, could have contributed to some extent to the global increase in asthma and allergic diseases.

### The variants of the hygiene hypothesis

As no specific type of infection was found to consistently support the hygiene hypothesis, some authors formulated more complex explanations.^44^^,^^45^ The ‘high turnover’ hypothesis proposed that, instead of a particular bacterial species or a stable colonization pattern, only a high turnover of different bacterial species and strains at the gut level would prevent atopy.^46^^,^^47^ The ‘old friends’ theory^48^ also named the ‘intracellular mild pathogens’ hypothesis^49^ suggested that increases in chronic inflammatory disorders in developed countries are partly attributable to diminishing exposure to organisms such as helminths and saprophytic mycobacteria that were immunologically tolerated as part of the mammalian evolutionary development.^50^ Finally, the ‘biodiversity hypothesis’ suggests that reduced biodiversity and alterations in the composition of the gut and skin microbiota are associated with various inflammatory conditions, including asthma, allergic and inflammatory bowel diseases, type 1 diabetes and obesity.^51–53^ Unfortunately, none of these hypotheses has provided consistent direct evidence that a generalized decrease in the exposure to microbial agents could be the main determinant of the asthma epidemic.

### Strengths and limitations of the hygiene hypothesis and its variants

A decade after its formulation, Strachan concluded that, despite remaining credible, the hygiene hypothesis requires more studies to unravel which infectious or other agents could play a protective effect on asthma.^34^ Today, thanks to its variants, the hygiene hypothesis still retains its appeal as a mechanistic explanation for the asthma epidemic. It offers a parsimonious, coherent and plausible explanation for the variations in allergy over time, between countries, between more and less affluent households, larger and smaller families and by position within the family. Its variants have expanded its biological plausibility and explained why in some particular populations asthma or/and allergy prevalence are particularly low or have been increasing. Importantly, given the steady moving from rural to urban environments of the worldwide population, it provides a unifying explanation for the parallel historical increases in asthma, allergic rhinitis, eczema and atopy as well as those in other autoimmune and inflammatory common diseases.^54^

By contrast, there is a considerable body of epidemiological evidence causing scepticism about the hygiene hypothesis (Table 2).^55^^,^^56^ First, results are inconsistent for every single microbial agent studied in relation to asthma. Second, none of the individual exposures of the hygiene hypothesis, such as family size^26^ and farming environments^37^^,^^42^ seems to explain a substantial proportion of the increase in asthma.^35^ Third, the hygiene hypothesis does not provide an explanation for the decline in asthma prevalence observed more recently in some Western countries.^55^^,^^57^ Fourth, the hypothesis does not explain the high asthma prevalence in Latin American cities^58^ and inner cities in the USA^59^ where poor hygiene could have played a protective effect on asthma. Fifth, the hygiene hypothesis implicitly refers to atopic asthma, but both atopic and non-atopic asthma have increased.^60^^,^^61^ Though not a scientific limitation, it has been suggested that its name conveys an outdated concept of hygiene and could equivocally suggest that less hygiene is better.^62^

**Table 2:** Relevant anomalies in the hygiene hypothesis as an explanation for the asthma epidemic

(i) Individual exposure to microbial agents has not been found to be consistently associated with asthma
(ii) Exposures consistently associated with asthma such as family size and farming do not explain or are unlikely to explain a significant proportion of asthma in the general population
(iii) The hygiene hypothesis does not provide an explanation for the decline in asthma prevalence observed more recently in some Western countries
(iv) The hypothesis does not explain the high asthma prevalence rates in cities in Latin America and inner cities in the USA
(v) The hygiene hypothesis relates to atopic asthma, but both atopic and non-atopic asthma appear to have been part of the epidemic increase
(vi) The hygiene hypothesis is likely to convey an outdated concept of hygiene and support the wrong notion that less hygiene is better

### The Westernization hypothesis

A different approach would be to focus on the exposures that have consistently been associated with the development of asthma that also fit the temporal and geographic patterns of the asthma epidemic. So far, observational studies have shown that parental asthma, environmental tobacco smoke in early life, including the prenatal period, and prematurity are well-established risk factors for childhood asthma.^63^ There is also evidence that modifiable behaviours or exposures during pregnancy (maternal weight gain or obesity, maternal use of antibiotics and maternal stress), the perinatal period (birth by Caesarean delivery) or post-natal life (severe Respiratory Syncytial Virus infection, overweight or obesity, indoor exposure to mould or fungi, and outdoor air pollution) may play a role.^63^^,^^64^ Regarding intervention studies, a recent Global Initiative for Asthma (GINA) report concluded that interventions aimed at reducing exposure to one single allergen (including house dust allergens) have not been effective, whereas multifaceted studies have shown some effectiveness that cannot be attributed to any single component.^65^ Other interventions with no evidence of preventing the occurrence of asthma included maternal dietary intake of fish, long-term polyunsaturated fatty acids or vitamin D during pregnancy.^65^

Considering the scarce and fragmented evidence of asthma aetiology, Douwes and Pearce suggested that ‘Westernization’ as a package of factors could explain the rise in asthma and allergic diseases worldwide.^61^ The literature about Westernization as related to the origin of asthma has included a wide range of environmental, social, behavioural and host factors and some specific agents (Table 3, references ^61^^,^^66–75^).

**Table 3:** Elements of the Westernization construct in papers related to the asthma epidemic

Factor	Exposure	Studies (Ref.)
Environmental and social factors	Increased urbanization	Murrison LB, 2019^66^; Weinberg EG, 2000^67^
	Time spent indoors	Murrison LB, 2019^66^
	Antibiotic use	Murrison LB, 2019^66^
	Air pollution	Murrison LB, 2019^66^
	Fungi/moulds	Murrison LB, 2019^66^
	Moisture damaged homes	Casas L, 2016^68^; Hahm MI, 2014^69^
	Sick building syndrome (newly built home)	Hahm MI, 2014^69^
	Infectious agents	Murrison LB, 2019^66^
	Smaller family size	Douwes J, 2002^61^
	Lack of farming	Casas L, 2016;^68^ Pechlivanis S, 2020^70^
	Travelling	Markevych I, 2017^95^
Behavioural factors	Tobacco smoke	Murrison LB, 2019^66^
	Physical activity	Murrison LB, 2019^66^
	Diet	Murrison LB, 2019^66^; Manzel A, 2014^71^; Litonjua AA, 2008^72^
	Maternal diet	Douwes J, 2002^61^
Host factors	Increased fetal growth	Douwes J, 2002^61^
	Obesity	Gibson PG, 2013^73^
	Caesarean delivery	Sevelsted A, 2015^74^
	Impaired skin barrier	Murrison LB, 2019^66^
Agents	Antibiotics	Douwes J, 2002^61^
	Vaccination	Douwes J, 2002^61^
	Chemicals	Murrison LB, 2019^66^
	Allergens	Murrison LB, 2019^66^
	Bacteria	Murrison LB, 2019^66^
	Virus	Murrison LB, 2019^66^
	Air pollutants	Murrison LB 2019^66^
	Vitamin D	Litonjua AA, 2007^72^
	Helminths (lack of exposure)	Smits HH, 2005^75^

**Table 4:** Main challenges that have limited the epidemiological approach to the asthma epidemic

(i) Relying on the assumption that the most likely explanation was a single cause
(ii) Asthma may not be a single homogeneous disease, but a number of different entities
(iii) Asthma could be part of a broader multimorbidity allergic syndrome
(iv) Chronic disease epidemiology is not well suited to understanding long life-course chronic diseases with remission and relapse periods
(v) For some potential risk factors, such as allergen exposure, reverse causation is hard to exclude

There are a number of strengths in the Westernization hypothesis. While still accommodating the hygiene hypothesis, it broadens the focus to factors such as nutrition or physical activity, which is something that could explain that the asthma epidemic had included both atopic and non-atopic asthma. Westernization is also an ongoing process that could span from changes that started in the nineteenth century to those still occurring in many places. The fact that in some places, some Westernizing factors are being substituted by more traditional ones could eventually explain that asthma and allergy are plateauing or decreasing. Since Westernization is a global construct, it offers more flexibility to accommodate the geographical and temporal heterogeneity of the asthma epidemic.^76^

On the negative side, most of the studies considering Westernization as an explanation for the asthma epidemic are rather narrative and vague, and do not formally elaborate on the construct of Westernization (Supplementary Table S1, available as Supplementary data at *IJE* online). Since Westernization includes a wide range of environmental and behavioural exposures, testing its aggregated causal influence in the asthma epidemic is a difficult task.

### Rethinking epidemiological research on the asthma epidemic

As none of the hypotheses posed so far has yet provided convincing evidence of the origins of the asthma epidemic, it seems appropriate to analyse the difficulties faced by epidemiological research in an attempt to understand its origin (Table 4 and Supplementary Table S2, available as Supplementary data at *IJE* online).

First, the predominant approach, particularly with regard to the hygiene hypothesis, has focused on single factors. This fits well with many epidemics, in particular epidemics of infectious diseases, but is difficult to adapt to most non-communicable diseases, including asthma, which are multifactorial.^77^ The Westernization hypothesis has suggested that testing the association between clusters of risk factors and asthma would yield more consistent results, as has been suggested for cardiovascular diseases.^78^ However, as far as we know, an assessment of a clustering pattern of risk factors in asthma has not been reported.

Second, our understanding of what is asthma may be insufficient, or even wrong. If asthma is an heterogenous collection of different diseases,^79^ then studies that group these conditions together will have difficulties in identifying the causal factors. Asthma has traditionally been classified into atopic and non-atopic asthma, or based on severity, control or age of onset. Increasingly, asthma phenotypes or endotypes are defined as TH2-high and TH2-low based on a combination of characteristics. For example, the multicentre ALLIANCE study used blood eosinophil counts and allergen-specific serum IgE antibodies to characterize participants with asthma. This study showed that asthmatics categorized as ‘atopy-only’, ‘eosinophils-only’, ‘T2-high’ (eosinophilia + atopy) and ‘T2-low’ (neither eosinophilia nor atopy) were distributed across all age groups, with those in the T2-high group showing more persistent asthma.^80^ A common strategy to deal with the heterogeneity of asthma is the use of composite phenotypes, such as atopic asthma.^37^ However, the latter has its own methodological problems as risk factors for atopy may be associated with atopic asthma, even in the absence of an effect on asthma^81^ (Supplementary material, available as Supplementary data at *IJE* online). Using the T2-high/T2-low phenotyping approach as used in the ALLIANCE study, preferably supplemented with sputum eosinophil measurements, may be more useful and could eventually result in more consistent associations with environmental exposures.

Third, asthma occurs in combination with other diseases, mainly with rhinoconjunctivitis and eczema, in a multimorbidity pattern.^82^^,^^83^ In fact, the asthma epidemic has been paralleled by an epidemic increase in allergic rhinitis and eczema.^22^^,^^23^ Thus, it could be that what we call an epidemic increase in asthma is indeed an epidemic increase in atopy and hay fever with a subsequent increase in asthma. This is supported by research suggesting that most of the increase in asthma is due to an increase in atopy and that the increase in asthma in non-atopic people was not accompanied by an increase in wheezing and could have been due to changes in reporting asthma.^84–86^ Studies suited to understanding the joint distribution of allergic phenotypes and their multimorbidity are likely to be more informative than studies addressing asthma as a single entity.

Fourth, canonical epidemiological methods may not be well suited to assessing the prevalence and etiological factors of diseases with an early-life origin and a long and complex life course such as asthma.^87^ Although asthma symptoms questionnaires validate well against clinical asthma diagnosis^88^ in prevalence studies, they are susceptible to self-reporting biases and cross-cultural differences in naming symptoms such as wheezing—something that could lead to prevalence figures higher than those observed in health record studies.^89^ However, the varying time course of asthma makes it difficult to classify incident cases, in particular when studying new-onset adult asthma,^90^ and to distinguish incidence from disease duration as determinants of prevalence. The latter is illustrated in the study of asthma among New Zealanders which suggested that the continued high prevalence of asthma in Māori adolescents and adults is probably due to a duration effect caused by inadequate access to healthcare, as well as exacerbating factors such as environmental tobacco smoke.^91^ To what extent the asthma epidemic has been the result of a genuine increase in incidence or by the contrary due to an increase in duration or both has not been formally assessed.

Fifth, epidemiological studies on asthma can be affected by reverse causation. It has been suggested that sensitization to allergens could be a consequence of asthma instead of a risk factor for asthma. In the case of obesity, for which there is consistent evidence of an association with asthma incidence,^92^^,^^93^ it has been shown that asthma is also associated with incident obesity, in both children and adults.^94^^,^^95^ Studies using Mendelian randomization support that obesity^96^ and BMI^97^ but not 25-hydroxyvitamin D^98^ are associated with asthma incidence.

Considering the intricacies in the epidemiological research of asthma alluded to above, it is no surprise that asthma is seen as a complex construct and that the hope that epidemiology alone can contribute to unravelling the origins of the asthma epidemic is called into question. Whether new studies on the hygiene-variants or Westernization hypotheses will ever resolve the enigma is hard to say but, in any case, a more systematic understanding of the different scientific challenges involved in this effort would be useful.

### Looking forward

Will it ever be possible to have a solid epidemiological explanation for why there was a global epidemic of asthma? As in the case of gastric ulcers and *Helicobacter pylori*,^99^ we must not exclude the possibility that the asthma epidemic is due to a single environmental or microbiological factor that has not yet been identified. If this were the case, the investigation of why, in some places and circumstances, environmental exposure on farms protects from the development of asthma and allergies could at some point lead to a relatively straightforward causal explanation for the asthma epidemic.

On the other hand, the origin of the asthma epidemic could be multifactorial and complex as suggested by the Westernization hypothesis. Current epidemiological methods are not well suited to addressing complex phenomena involving non-linearity and ‘feedback loops’, though, if properly addressed, the epidemiological study of this complexity could produce findings that are more specific to the population under study and potentially be of direct public health relevance and validity.^100^ One way to study complex health patterns is the use of systems science models, which so far have been mostly applied to test the impact of interventions and to support policy modelling.^101^ Applying this type of models to understand the mechanisms and feedback processes that give rise to the population distribution of health outcomes, such as the asthma epidemic, is an underexplored field.^101^ Recent studies have used systems science models to understand the causal dynamics of obesity^102^ and opioid addiction^103^—something that has stimulated a dialog between canonical epidemiology and the systems science approach.^101^^,^^104^ Another alternative to consider is the use of complex simulation with agent-based models^105^ and synthetic populations.^106^ Although these types of complex models have been used in different contexts, such as exploring geographical patterns at the neighbourhood level or in policy development, it could be useful to explore whether they could also help in understanding the origins of the asthma epidemic.

The use of systems science methods to understand the asthma epidemic as suggested above may involve the need to approach some specific intricate issues such as the complexity of the causal chain, the heterogeneity of the clinical phenotypes and the existence of age-cohort-period effects. Regarding the causal chain, though mostly used in genetic or molecular studies, methods such as the Markov-chain models^107^ could help to unravel complex interactions of causal factors in asthma or model state transitions such as those from allergen exposure to sensitization to clinical expressions, and to generate hypotheses for more refined simulation models. A recent study using an exposome approach has shown that some profiles, as combinations of risk factors, were associated with asthma in a French population cohort.^108^ Regarding clinical heterogeneity, Markov-chain models could facilitate a better understanding of the complex incidence–remitting–relapsing behaviour of asthma.^109^ In the case of asthma multimorbidity, the recent use of machine-learning methods^82^^,^^83^ could be eventually extended with the simulation of synthetic populations.

Revisiting the asthma epidemic with a complex systems approach could offer a unique opportunity to formulate new questions and research frames, and would require a concerted interdisciplinary effort. Epidemiology has played a major role in our understanding of asthma, but asthma epidemiology alone is unlikely to solve the type of scientific hurdles that research on the asthma epidemic has encountered. Neither are basic laboratory science (which too often is wedded to an allergic paradigm) or clinical research likely to solve these problems alone.

## Ethics approval

Not needed according to *IJE* author guidelines.

## Supplementary Material

dyad035_Supplementary_Data

## Data Availability

Not applicable in accordance with *IJE* author guidelines.

## References

[1] Burr ML , Butland BK, King S, Vaughan-Williams E. Changes in asthma prevalence: two surveys 15 years apart. Arch Dis Child 1989;64:1452–56. 2817930 10.1136/adc.64.10.1452 PMC1792778

[2] Woolcock AJ , Peat JK. Evidence for the increase in asthma worldwide. Ciba Found Symp 1997;206:122–34. 9257009 10.1002/9780470515334.ch8

[3] Doll R , Hill AB. Lung cancer and other causes of death in relation to smoking. Br Med J 1956;Nov 102:1071–81. 13364389 10.1136/bmj.2.5001.1071 PMC2035864

[4] Kannel WB , McGee D, Gordon T. A general cardiovascular risk profile: the Framingham Study. Am J Cardiol 1976; 38:46–51. 132862 10.1016/0002-9149(76)90061-8

[5] Pearce N , Crane J, Burgess C, Beasley R, Jackson R. Fenoterol and asthma mortality. Lancet 1989;1:1196–97. 2566750 10.1016/s0140-6736(89)92768-2

[6] Antó JM , Sunyer J, Rodriguez-Roisin R, Suarez-Cervera M, Vazquez L. Community outbreaks of asthma associated with inhalation of soybean dust: Toxicoepidemiological Committee. N Engl J Med 1989;320:1097–102. 2710172 10.1056/NEJM198904273201701

[7] Eder W , Ege MJ, von Mutius E. The asthma epidemic. N Engl J Med 2006;355:2226–35. 17124020 10.1056/NEJMra054308

[8] Latvala J , von Hertzen L, Lindholm H, Haahtela T. Trends in prevalence of asthma and allergy in Finnish young men: nationwide study. BMJ 2005;330:1186–87. 15849204 10.1136/bmj.38448.603924.AE PMC558015

[9] Deckers IAG , McLean S, Linssen S, Mommers M, van Schayck CP, Sheikh A. Investigating international time trends in the incidence and prevalence of atopic eczema 1990-2010: a systematic review of epidemiological studies. PLoS One 2012;7:e39803. 22808063 10.1371/journal.pone.0039803 PMC3394782

[10] Burney P , Chinn S, Luczynska C et al Variations in the prevalence of respiratory symptoms, self-reported asthma attacks, and use of asthma medication in the European Community Respiratory Health Survey (ECRHS). Eur Respir J 1996;9:687–95. 8726932 10.1183/09031936.96.09040687

[11] Chinn S , Burney P, Jarvis D, Luczynska C. Variation in bronchial responsiveness in the European Community Respiratory Health Survey (ECRHS). Eur Respir J 1997;10:2495–501. 9426085 10.1183/09031936.97.10112495

[12] Asher MI , Keil U, Anderson HR et al International Study of Asthma and Allergies in Childhood (ISAAC): rationale and methods. Eur Respir J 1995;8:483–91. 7789502 10.1183/09031936.95.08030483

[13] Asher MI , Anderson HR, Stewart AW, Crane J. Worldwide variations in the prevalence of asthma symptoms: the International Study of Asthma and Allergies in Childhood (ISAAC). Eur Respir J 1998;12:315–35. 9727780 10.1183/09031936.98.12020315

[14] Pearce N , Sunyer J, Cheng S et al Comparison of asthma prevalence in the ISAAC and the ECRHS. ISAAC Steering Committee and the European Community Respiratory Health Survey. International Study of Asthma and Allergies in Childhood. Eur Respir J 2000;16:420–26. 11028654 10.1183/9031936.00.16337700

[15] Wong GWK , Ko FWS, Hui DSC et al Factors associated with difference in prevalence of asthma in children from three cities in China: multicentre epidemiological survey. BMJ 2004;329:486. 15331473 10.1136/bmj.329.7464.486 PMC515199

[16] von Mutius E , Martinez FD, Fritzsch C, Nicolai T, Roell G, Thiemann HH. Prevalence of asthma and atopy in two areas of West and East Germany. Am J Respir Crit Care Med 1994;149:358–64. 8306030 10.1164/ajrccm.149.2.8306030

[17] Pekkarinen PT , von Hertzen L, Laatikainen T et al A disparity in the association of asthma, rhinitis, and eczema with allergen-specific IgE between Finnish and Russian Karelia. Allergy 2007;62:281–87. 17298345 10.1111/j.1398-9995.2006.01249.x

[18] Perzanowski MS , Ng’ang’a LW, Carter MC et al Atopy, asthma, and antibodies to Ascaris among rural and urban children in Kenya. J Pediatr 2002;140:582–88. 12032526 10.1067/mpd.2002.122937

[19] Priftanji A , Strachan D, Burr M et al Asthma and allergy in Albania and the UK. Lancet 2001;358:1426–27. 11705492 10.1016/S0140-6736(01)06521-7

[20] Chinn S , Jarvis D, Burney P et al Increase in diagnosed asthma but not in symptoms in the European Community Respiratory Health Survey. Thorax 2004;59:646–51. 15282382 10.1136/thx.2004.021642 PMC1747094

[21] Chinn S , Downs SH, Anto JM et al; SAPALDIA. Incidence of asthma and net change in symptoms in relation to changes in obesity. Eur Respir J 2006;28:763–71. 16870655 10.1183/09031936.06.00150505

[22] Asher MI , Montefort S, Björkstén B et al Worldwide time trends in the prevalence of symptoms of asthma, allergic rhinoconjunctivitis, and eczema in childhood: ISAAC Phases One and Three repeat multicountry cross-sectional surveys. Lancet 2006;368:733–43. 16935684 10.1016/S0140-6736(06)69283-0

[23] Laatikainen T , von Hertzen L, Koskinen J-P et al Allergy gap between Finnish and Russian Karelia on increase. Allergy 2011;66:886–92. 21255037 10.1111/j.1398-9995.2010.02533.x

[24] Barker DJ. Acute appendicitis and dietary fibre: an alternative hypothesis. Br Med J (Clin Res Ed) 1985;290:1125–27. 10.1136/bmj.290.6475.1125 PMC1418708 2985169

[25] Morris J , Barker DJ, Nelson M. Diet, infection, and acute appendicitis in Britain and Ireland. J Epidemiol Community Health 1987;41:44–49. 3668458 10.1136/jech.41.1.44 PMC1052575

[26] Strachan DP. Hay fever, hygiene, and household size. BMJ 1989;299:1259–60. 2513902 10.1136/bmj.299.6710.1259 PMC1838109

[27] Busse WW , Lemanske RF. Asthma. N Engl J Med 2001;344:350–62. 11172168 10.1056/NEJM200102013440507

[28] Arrais M , Maricoto T, Cooper P et al Helminth infections, atopy, asthma and allergic diseases: protocol for a systematic review of observational studies worldwide. BMJ Open 2020;10:e038085. 10.1136/bmjopen-2020-038085 PMC7252955 32457081

[29] Arnoldussen DL , Linehan M, Sheikh A. BCG vaccination and allergy: a systematic review and meta-analysis. J Allergy Clin Immunol 2011;127:246–53, 253.e1–21. 20933258 10.1016/j.jaci.2010.07.039

[30] Matricardi PM , Rosmini F, Ferrigno L et al Cross sectional retrospective study of prevalence of atopy among Italian military students with antibodies against hepatitis A virus. BMJ 1997;314:999–1003. 9112843 10.1136/bmj.314.7086.999 PMC2126410

[31] Jarvis D , Luczynska C, Chinn S, Burney P. The association of hepatitis A and Helicobacter pylori with sensitization to common allergens, asthma and hay fever in a population of young British adults. Allergy 2004;59:1063–67. 15355464 10.1111/j.1398-9995.2004.00539.x

[32] Nagel G , Weinmayr G, Flohr C, Kleiner A, Strachan DP; ISAAC Phase Two Study Group. Association of pertussis and measles infections and immunizations with asthma and allergic sensitization in ISAAC Phase Two. Pediatr Allergy Immunol 2012;23:737–46. 23005697 10.1111/pai.12007

[33] Murk W , Risnes KR, Bracken MB. Prenatal or early-life exposure to antibiotics and risk of childhood asthma: a systematic review. Pediatrics 2011;127:1125–38. 21606151 10.1542/peds.2010-2092

[34] Strachan DP. Family size, infection and atopy: the first decade of the ‘hygiene hypothesis’. Thorax 2000;55(Suppl 1): S2–10. 10943631 10.1136/thorax.55.suppl_1.s2 PMC1765943

[35] Wickens K , Crane J, Pearce N, Beasley R. The magnitude of the effect of smaller family sizes on the increase in the prevalence of asthma and hay fever in the United Kingdom and New Zealand. J Allergy Clin Immunol 1999;104:554–58. 10482827 10.1016/s0091-6749(99)70323-4

[36] Ball TM , Castro-Rodriguez JA, Griffith KA, Holberg CJ, Martinez FD, Wright AL. Siblings, day-care attendance, and the risk of asthma and wheezing during childhood. N Engl J Med 2000;343:538–43. 10954761 10.1056/NEJM200008243430803

[37] Braun-Fahrländer C , Riedler J, Herz U et al Environmental exposure to endotoxin and its relation to asthma in school-age children. N Engl J Med 2002;347:869–77. 12239255 10.1056/NEJMoa020057

[38] von Mutius E. 99th Dahlem conference on infection, inflammation and chronic inflammatory disorders: farm lifestyles and the hygiene hypothesis. Clin Exp Immunol 2010;160:130–35. 20415863 10.1111/j.1365-2249.2010.04138.x PMC2841847

[39] Douwes J , Travier N, Huang K et al Lifelong farm exposure may strongly reduce the risk of asthma in adults. Allergy 2007;62:1158–65. 17845585 10.1111/j.1398-9995.2007.01490.x

[40] Douwes J , Boezen M, Brooks C, Pearce N, Chronic obstructive pulmonary disease and asthma. In: Detels R, Gulliford M, Karim QA, Tan CC (eds). Oxford Textbook of Global Public Health. Oxford University Press, 2015:945–69.

[41] Stein MM , Hrusch CL, Gozdz J et al Innate immunity and asthma risk in Amish and Hutterite farm children. N Engl J Med 2016;375:411–21. 27518660 10.1056/NEJMoa1508749 PMC5137793

[42] Sozańska B , Błaszczyk M, Pearce N, Cullinan P. Atopy and allergic respiratory disease in rural Poland before and after accession to the European Union. J Allergy Clin Immunol 2014;133:1347–53. 24342546 10.1016/j.jaci.2013.10.035

[43] Melsom T , Brinch L, Hessen JO et al Asthma and indoor environment in Nepal. Thorax 2001;56:477–81. 11359965 10.1136/thorax.56.6.477 PMC1746067

[44] Sepp E , Julge K, Vasar M, Naaber P, Björksten B, Mikelsaar M. Intestinal microflora of Estonian and Swedish infants. Acta Paediatr 1997;86:956–61. 9343275 10.1111/j.1651-2227.1997.tb15178.x

[45] Björkstén B , Naaber P, Sepp E, Mikelsaar M. The intestinal microflora in allergic Estonian and Swedish 2-year-old children. Clin Exp Allergy 1999;29:342–46. 10202341 10.1046/j.1365-2222.1999.00560.x

[46] Wold AE. The hygiene hypothesis revised: is the rising frequency of allergy due to changes in the intestinal flora? Allergy 1998;53:20–25. 10.1111/j.1398-9995.1998.tb04953.x 9825991

[47] Matricardi PM , Bonini S. High microbial turnover rate preventing atopy: a solution to inconsistencies impinging on the hygiene hypothesis? Clin Exp Allergy 2000;30:1506–10. 11069557 10.1046/j.1365-2222.2000.00994.x

[48] Rook G , Stanford JL. Give us this day our daily germs. Immunol Today 1998;19:113–16. 9540269 10.1016/s0167-5699(97)01204-8

[49] Rook G. 99th Dahlem conference on infection, inflammation and chronic inflammatory disorders: Darwinian medicine and the ‘hygiene’ or ‘old friends’ hypothesis. Clin Exp Immunol 2010;160:70–79. 20415854 10.1111/j.1365-2249.2010.04133.x PMC2841838

[50] Rook G. The hygiene hypothesis and the increasing prevalence of chronic inflammatory disorders. Trans R Soc Trop Med Hyg 2007;101:1072–74. 17619029 10.1016/j.trstmh.2007.05.014

[51] von HL , Hanski I, Haahtela T. Natural immunity: biodiversity loss and inflammatory diseases are two global megatrends that might be related. EMBO Rep 2011;12:1089–93. 21979814 10.1038/embor.2011.195 PMC3207110

[52] Haahtela T , Holgate S, Pawankar R et al; WAO Special Committee on Climate Change and Biodiversity. The biodiversity hypothesis and allergic disease: world allergy organization position statement. World Allergy Organ J 2013;6:3. 23663440 10.1186/1939-4551-6-3 PMC3646540

[53] Haahtela T. A biodiversity hypothesis. Allergy 2019;74:1445–56. 30835837 10.1111/all.13763

[54] Bach J-F. The effect of infections on susceptibility to autoimmune and allergic diseases. N Engl J Med 2002;347:911–20. 12239261 10.1056/NEJMra020100

[55] Douwes J , Pearce N. Commentary: The end of the hygiene hypothesis? Int J Epidemiol 2008;37:570–72. 18456712 10.1093/ije/dyn077

[56] Brooks C , Pearce N, Douwes J. The hygiene hypothesis in allergy and asthma: an update. Curr Opin Allergy Clin Immunol 2013;13:70–77. 23103806 10.1097/ACI.0b013e32835ad0d2

[57] Ramsey CD , Celedón JC. The hygiene hypothesis and asthma. Curr Opin Pulm Med 2005;11:14–20. 15591883 10.1097/01.mcp.0000145791.13714.ae

[58] Cooper PJ , Rodrigues LC, Barreto ML. Influence of poverty and infection on asthma in Latin America. Curr Opin Allergy Clin Immunol 2012;12:171–78. 22391754 10.1097/ACI.0b013e3283510967 PMC7612855

[59] Eggleston PA , Buckley TJ, Breysse PN, Wills-Karp M, Kleeberger SR, Jaakkola JJ. The environment and asthma in U.S. inner cities. Environ Health Perspect 1999;107(Suppl 3):439–50. 10346992 10.1289/ehp.99107s3439 PMC1566223

[60] Thomsen SF , Ulrik CS, Larsen K, Backer V. Change in prevalence of asthma in Danish children and adolescents. Ann Allergy Asthma Immunol 2004;92:506–11. 15191018 10.1016/S1081-1206(10)61757-7

[61] Douwes J , Pearce N. Asthma and the Westernization ‘package’. Int J Epidemiol 2002;31:1098–102. 12540698 10.1093/ije/31.6.1098

[62] Bloomfield SF , Rook GA, Scott EA, Shanahan F, Stanwell-Smith R, Turner P. Time to abandon the hygiene hypothesis: new perspectives on allergic disease, the human microbiome, infectious disease prevention and the role of targeted hygiene. Perspect Public Health 2016;136:213–24. 27354505 10.1177/1757913916650225 PMC4966430

[63] Institute of Medicine (US) Committee on the Assessment of Asthma and Indoor Air. Clearing the Air: Asthma and Indoor Air Exposures. Washington, DC: National Academies Press (US), 2000. 25077220

[64] Castro-Rodriguez JA , Forno E, Rodriguez-Martinez CE, Celedón JC. Risk and protective factors for childhood asthma: what is the evidence? J Allergy Clin Immunol Pract 2016;4:1111–22. 27286779 10.1016/j.jaip.2016.05.003 PMC5107168

[65] GINA. *Global Initiative for Asthma – Global Strategy for Asthma Management and Prevention*, 2022. www.ginasthma.org (7 March 2023, date last accessed).

[66] Murrison LB , Brandt EB, Myers JB, Hershey GKK. Environmental exposures and mechanisms in allergy and asthma development. J Clin Invest 2019;129:1504–15. 30741719 10.1172/JCI124612 PMC6436881

[67] Weinberg EG. Urbanization and childhood asthma: an African perspective. J Allergy Clin Immunol 2000;105:224–31. 10669840 10.1016/s0091-6749(00)90069-1

[68] Casas L , Tischer C, Täubel M. Pediatric asthma and the indoor microbial environment. Curr Environ Health Rep 2016;3:238–49. 27230430 10.1007/s40572-016-0095-y

[69] Hahm M-I , Chae Y, Kwon H-J et al Do newly built homes affect rhinitis in children? The ISAAC phase III study in Korea. Allergy 2014;69:479–87. 24428419 10.1111/all.12355

[70] Pechlivanis S , von Mutius E. Effect of farming on asthma. Acta Med Acad 2020;49:144–55. 33189120 10.5644/ama2006-124.293

[71] Manzel A , Muller DN, Hafler DA, Erdman SE, Linker RA, Kleinewietfeld M. Role of ‘Western diet’ in inflammatory autoimmune diseases. Curr Allergy Asthma Rep 2014;14:404. 24338487 10.1007/s11882-013-0404-6 PMC4034518

[72] Litonjua AA. Dietary factors and the development of asthma. Immunol Allergy Clin North Am 2008;28:603–29.ix. 18572110 10.1016/j.iac.2008.03.005 PMC2536613

[73] Gibson PG. Obesity and asthma. Ann Am Thorac Soc 2013;10:S138–42. 24313764 10.1513/AnnalsATS.201302-038AW

[74] Sevelsted A , Stokholm J, Bønnelykke K, Bisgaard H. Cesarean section and chronic immune disorders. Pediatrics 2015;135:e92–98. 25452656 10.1542/peds.2014-0596

[75] Smits HH , Hartgers FC, Yazdanbakhsh M. Helminth infections: protection from atopic disorders. Curr Allergy Asthma Rep 2005;5:42–50. 15659262 10.1007/s11882-005-0053-5

[76] Matricardi PM. 99th Dahlem conference on infection, inflammation and chronic inflammatory disorders: controversial aspects of the ‘hygiene hypothesis’. Clin Exp Immunol 2010;160:98–105. 20415858 10.1111/j.1365-2249.2010.04130.x PMC2841842

[77] Pearce N. Epidemiology in a changing world: variation, causation and ubiquitous risk factors. Int J Epidemiol 2011;40:503–12. 21247886 10.1093/ije/dyq257

[78] Andersen LB , Boreham CAG, Young IS et al Insulin sensitivity and clustering of coronary heart disease risk factors in young adults. Prev Med 2006;42:73–77. 16330091 10.1016/j.ypmed.2005.10.009

[79] Pavord ID , Beasley R, Agusti A et al After asthma: redefining airways diseases. Lancet 2018;391:350–400. 28911920 10.1016/S0140-6736(17)30879-6

[80] Maison N , Omony J, Illi S et al T2-high asthma phenotypes across lifespan. Eur Respir J 2022;60:2102288. 35210326 10.1183/13993003.02288-2021 PMC9520028

[81] Richiardi L , Barone-Adesi F, Pearce N. Cancer subtypes in aetiological research. Eur J Epidemiol 2017;32:353–61. 28497292 10.1007/s10654-017-0253-z

[82] Pinart M , Benet M, Annesi-Maesano I et al Comorbidity of eczema, rhinitis, and asthma in IgE-sensitised and non-IgE-sensitised children in MeDALL: a population-based cohort study. Lancet Respir Med 2014;2:131–40. 24503268 10.1016/S2213-2600(13)70277-7

[83] Garcia-Aymerich J , Benet M, Saeys Y et al Phenotyping asthma, rhinitis and eczema in MeDALL population-based birth cohorts: an allergic comorbidity cluster. Allergy 2015;70:973–84. 25932997 10.1111/all.12640

[84] Burney P. The changing prevalence of asthma? Thorax 2002;57:ii36–39. 12364709 PMC1766004

[85] Upton MN , McConnachie A, McSharry C et al Intergenerational 20 year trends in the prevalence of asthma and hay fever in adults: the Midspan family study surveys of parents and offspring. BMJ 2000;321:88–92. 10884260 10.1136/bmj.321.7253.88 PMC27429

[86] Kuehni CE , Davis A, Brooke AM, Silverman M. Are all wheezing disorders in very young (preschool) children increasing in prevalence? Lancet 2001;357:1821–25. 11410189 10.1016/S0140-6736(00)04958-8

[87] Antó JM. The causes of asthma: the need to look at the data with different eyes. Allergy 2004;59:121–23. 14763923 10.1111/j.1398-9995.2003.00480.x

[88] Pekkanen J , Pearce N. Defining asthma in epidemiological studies. Eur Respir J 1999;14:951–57. 10573248 10.1034/j.1399-3003.1999.14d37.x

[89] Bloom CI , Saglani S, Feary J, Jarvis D, Quint JK. Changing prevalence of current asthma and inhaled corticosteroid treatment in the UK: population-based cohort 2006-2016. Eur Respir J 2019;53:1802130. 30765507 10.1183/13993003.02130-2018

[90] Antó JM , Sunyer J, Basagaña X et al Risk factors of new-onset asthma in adults: a population-based international cohort study. Allergy 2010;65:1021–30. 20132157 10.1111/j.1398-9995.2009.02301.x

[91] Pomare E , Tutengaehe H, Ramsden I, Hight M, Pearce N, Ormsby V. Asthma in Maori people. N Z Med J 1992;105:469–70. 1436873

[92] Ford ES , Mannino DM, Redd SC, Mokdad AH, Mott JA. Body mass index and asthma incidence among USA adults. Eur Respir J 2004;24:740–44. 15516666 10.1183/09031936.04.00088003

[93] Ali Z , Ulrik CS. Obesity and asthma: a coincidence or a causal relationship? A systematic review. Respir Med 2013;107:1287–300. 23642708 10.1016/j.rmed.2013.03.019

[94] Contreras ZA , Chen Z, Roumeliotaki T et al Does early onset asthma increase childhood obesity risk? A pooled analysis of 16 European cohorts. Eur Respir J 2018;52:1800504. 30209194 10.1183/13993003.00504-2018 PMC6443037

[95] Moitra S , Carsin A-E, Abramson MJ et al Long-term effect of asthma on the development of obesity among adults: an international cohort study, ECRHS. Thorax 2023;78:128–35. 35477559 10.1136/thoraxjnl-2021-217867

[96] Sun Y-Q , Brumpton BM, Langhammer A, Chen Y, Kvaløy K, Mai X-M. Adiposity and asthma in adults: a bidirectional Mendelian randomisation analysis of The HUNT Study. Thorax 2020;75:202–08. 31611343 10.1136/thoraxjnl-2019-213678

[97] Skaaby T , Taylor AE, Thuesen BH et al Estimating the causal effect of body mass index on hay fever, asthma and lung function using Mendelian randomization. Allergy 2018;73:153–64. 28675761 10.1111/all.13242

[98] Manousaki D , Paternoster L, Standl M et al Vitamin D levels and susceptibility to asthma, elevated immunoglobulin E levels, and atopic dermatitis: a Mendelian randomization study. PLoS Med 2017;14:e1002294. 28486474 10.1371/journal.pmed.1002294 PMC5423551

[99] Marshall B. Helicobacter pylori: 20 years on. Clin Med (Lond) 2002;2:147–52. 11991099 10.7861/clinmedicine.2-2-147 PMC4952378

[100] Pearce N , Merletti F. Complexity, simplicity, and epidemiology. Int J Epidemiol 2006;35:515–19. 16415326 10.1093/ije/dyi322

[101] Cerdá M , Keyes KM. Systems modeling to advance the promise of data science in epidemiology. Am J Epidemiol 2019;188:862–65. 30877289 10.1093/aje/kwy262 PMC6494667

[102] Gittner LS , Kilbourne BJ, Vadapalli R, Khan HMK, Langston MA. A multifactorial obesity model developed from nationwide public health exposome data and modern computational analyses. Obes Res Clin Pract 2017;11:522–33. 28528799 10.1016/j.orcp.2017.05.001 PMC5597453

[103] Galea S , Hall C, Kaplan GA. Social epidemiology and complex system dynamic modelling as applied to health behaviour and drug use research. Int J Drug Policy 2009;20:209–16. 18930649 10.1016/j.drugpo.2008.08.005 PMC2782722

[104] Galea S , Hernán MA. Galea and Hernán respond to brings to the table,’ ‘differential measurement error,’ and ‘causal inference in social epidemiology’. Am J Epidemiol 2020;189:183–84. 31566213 10.1093/aje/kwz201 PMC7217273

[105] Orr MG , Kaplan GA, Galea S. Neighbourhood food, physical activity, and educational environments and black/white disparities in obesity: a complex systems simulation analysis. J Epidemiol Community Health 2016;70:862–67. 27083491 10.1136/jech-2015-205621

[106] Knight J , Wells S, Marshall R, Exeter D, Jackson R. Developing a synthetic national population to investigate the impact of different cardiovascular disease risk management strategies: a derivation and validation study. PLoS One 2017;12:e0173170. 28384217 10.1371/journal.pone.0173170 PMC5383032

[107] Baurley JW , Conti DV, Gauderman WJ, Thomas DC. Discovery of complex pathways from observational data. Stat Med 2010;29:1998–2011. 20683892 10.1002/sim.3962 PMC2922970

[108] Guillien A , Bédard A, Dumas O et al Exposome profiles and asthma among French adults. Am J Respir Crit Care Med 2022;206:1208–19. 35816632 10.1164/rccm.202205-0865OC

[109] Albert PS. A Markov model for sequences of ordinal data from a relapsing-remitting disease. Biometrics 1994;50:51–60. 8086615

