# Supplementary material for: Why has epidemiology not (yet) succeeded in identifying the origin of the asthma epidemic?
Source: Int J Epidemiol. 2023 Apr 2;52(4):974–83. doi: 10.1093/ije/dyad035 (PMC10396414; doi:10.1093/ije/dyad035)
Supplement: dyad035_Supplementary_Data [file dyad035_supplementary_data.zip › dyad035_Supplementary_Data/ije-2022-09-1126-File003.docx]

**On line supplement**

**Table S1. Narrative constructs of westernization in papers related to the asthma epidemic.**

1. Douwes J, Pearce N. Asthma and the westernization ‘package’. Int J Epidemiol. 2002 Dec 1;31(6):1098–1102. “Thus, it is important that we consider the forest of changes that occur with westernization, as well as doing studies of specific trees”
2. 1. Douwes J, Pearce N. Asthma and the westernization ‘package’. Int J Epidemiol. 2002 Dec 1;31(6):1098–1102. “It is likely that the package is more than the sum of its parts, and that these social and environmental changes are all pushing infant’s immune system in the same direction. To know what the direction is, and which components of the package are responsible, requires that better etiological theories of asthma are developed to replace the allergen theory, or to incorporate it as a special case”
3. Gibson PG. Obesity and asthma. Ann Am Thorax Soc 2013 Dec;**10** Suppl: S138-42. “There is a global epidemic of asthma and obesity that is concentrated in Westernized and developed countries”.
4. Casas L, Tischer C, Täubel M. Pediatric Asthma and the Indoor Microbial Environment. Review - Curr Environ Health Rep. 2016 Sep;**3**(3):238-49. “The global increase in the prevalence of asthma has been related to several risk factors; many of them linked to the "westernization" process and the characteristics of the indoor microbial environment during early life may play an important role”
5. Sevelsted A, Stokholm J, Bønnelykke K, et al. Pediatrics. 2015 Jan;**135**(1):e92-8. “Immune diseases such as asthma, allergy, inflammatory bowel disease, and type 1 diabetes have shown a parallel increase in prevalence during recent decades in westernized countries. The rate of cesarean delivery has also increased in this period and has been associated with the development of some of these diseases”.
6. Litonjua AA. Dietary factors and the development of asthma. Review- Immunol Allergy Clin North Am 2008 Aug;**28**(3):603-29. “Asthma and allergies continue to be major public health problems in affluent westernized countries. Changes in dietary habits dating from before the increase in asthma prevalence may have a role in the development of asthma and allergies”
7. Cooper PJ, Rodrigues LC, Cruz AA et al. Asthma in Latin America: a public heath challenge and research opportunity. Review Allergy. 2009 Jan;**64**(1):5-17. “The causes of asthma in Latin America are likely to be associated with urbanization, migration, and the adoption of a modern 'Westernized' lifestyle and environmental changes that follow these processes that include changes in diet, physical activity, hygiene, and exposures to allergens, irritants, and outdoor and indoor pollutants”.
8. Pechlivanis S, von Mutius E, Effect of Farming on Asthma. Review - Acta Med Acad 2020 Aug;**49**(2):144-155. “The reasons for the increase in the prevalence of asthma worldwide is still unclear but has been hypothesized to be attributable to westernization/urbanization of rural regions thus resulting in the loss of rural farming environmental.
9. Weinberg EG. Urbanization and childhood asthma: an African perspective. Review- J Allergy Clin Immunol. 2000 Feb;**105**(2 Pt 1):224-31. “A so-called Western lifestyle has been the factor most commonly cited to explain this worrying increase in asthma prevalence. In essence, this implies a way of life where children are exposed from early infancy to a wide range of foods, infections, indoor and outdoor allergens, and irritants and to the effects of motor vehicle pollution. Until fairly recently, children in many African countries lived mainly in rural areas and were not exposed to the effects of a Western lifestyle” .
10. Litounja AA, Weiss ST. Is vitamin D deficiency to blame for the asthma epidemic? J Allergy Clin Immunol. 2007 Nov;**120**(5):1031-5. “We hypothesize that as populations grow more prosperous, more time is spent indoors, and there is less exposure to sunlight, leading to decreased cutaneous vitamin D production. Vitamin D deficiency has been associated with obesity, African American race (particularly in urban, inner-city settings), and recent immigrants to westernized countries, thus reflecting the epidemiologic patterns observed in the asthma epidemic”.
11. Hahm MI, Chae Y, Kwon HJ et al. Do newly built homes affect rhinitis in children? The ISAAC phase III study in Korea. Allergy 2014. Apr;**69**(4):479-87. “As Korea has experienced rapid economic development, the lifestyles of Koreans have become more Westernized, in terms of urbanization and dietary habits. Also, the prevalences of allergic diseases, such as rhinitis, asthma, and dermatitis, have increased rapidly” .
12. Dowse GK, Smith D, Turner KJ et al. Prevalence and features of asthma in a sample survey of urban Goroka, Papua New Guinea. Clinical Allergy, 01 Sep 1985. **15**(5):429-438. “The implication is that particular local aetiological factors may be responsible for the documented rise in prevalence in the South Fore (Okapa) people, rather than the process of 'Westernization' per se”.
13. Smits HH, Hartgers FC, Yazdanbakhsh M. Helminth infections: protection from atopic disorders. Review Curr Allergy Asthma Rep 2005. Jan;**5**(1):42-50. “Westernized countries are suffering from an epidemic rise in immunologic disorders, such as childhood allergy. A popular explanation is that the increased prevalence in allergy is due to a diminished or altered exposure to gut-dwelling microbes, resulting in a disordered immunoregulation”.
14. Markevych I, Baumbach C, Standl M et al. Early life travelling does not increase risk of atopic outcomes until 15 years: results from GINIplus and LISAplus. Clin Exp Allergy. 2017 Mar;**47**(3):395-400 “Westernized lifestyle has been blamed for allergy epidemics. One of its characteristics is increased distances and frequency of travelling from early life onwards. Early life travelling to places which substantially differ from home environment in terms of climate, vegetation and food could increase the exposure to further unknown allergens and hence promote the development of allergies, but no epidemiological study has investigated this speculation”.

**Table S2. Gaps in the epidemiological research of the asthma epidemic**

1. Lack of studies focusing on the joint distribution of all allergic diseases instead of one single disease.
2. Lack of annual surveys in the same areas to monitor incidence, remission and relapse at a small geographical resolution.
3. Lack of frequently repeated measurements of biological markers of asthma, mainly BRH.
4. Limited and delayed assessment of the attributable fraction of the candidate exposures like in family size.
5. Limited attention to multimorbidity of allergic diseases, mainly asthma, allergic rhinitis and eczema as well as other immune related diseases.
6. Common use of compounded phenotypes leading to ambiguity in the attribution of risk factors.
7. Insufficient theoretical work on multifactorial epidemics including assessment of risk factors clustering and its attributable fractions.
8. Lack of formal constructs and approaches to understand the effects of westernization and globalization.
