# Supplementary material for: Why has epidemiology not (yet) succeeded in identifying the origin of the asthma epidemic?
Source: Int J Epidemiol. 2023 Apr 2;52(4):974–83. doi: 10.1093/ije/dyad035 (PMC10396414; doi:10.1093/ije/dyad035)
Supplement: dyad035_Supplementary_Data [file dyad035_supplementary_data.zip › dyad035_Supplementary_Data/ije-2022-09-1126-File004.docx]

**On line Supplement: Disease phenotypes and pathogenic mechanisms**

**Richiardi L, Pearce N**

It is widely recognized that, in the context of aetiological research, disease phenotypes defined on the basis of combinations of selected characteristics, including symptoms, molecular patterns, and morphological features, have aetiological value when they are markers of distinct pathogenic mechanisms. However, as Richiardi et al [ref] show, when these characteristics are not part of the causal pathway, then using them to define disease phenotypes can produce invalid or misleading findings. We use the terminology of Richiardi et al here, and apply it to several different possible scenarios relating to asthma phenotypes.

***Figure S1.*** *Simplified diagram showing the relationship between exposures (E), pathogenic mechanisms (A), the disease (Asthma), characteristics (C) and phenotypes.*

**
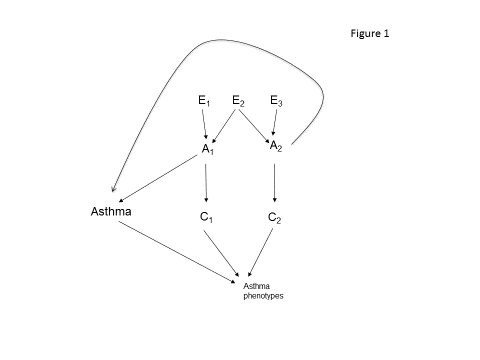
**

Figure S1 summarizes the supposed relationships between a disease entity, its phenotypes and the associated pathogenic mechanisms: E denotes an exposure, A denotes a pathogenic mechanism (e.g. an allergic mechanism) leading to a disease (asthma), and C is a characteristic (e.g. skin-prick test positivity) that is used alone or in combination with other characteristics to define an asthma phenotype (Ph). All the pathogenic mechanisms (A1, A2) lead to the diagnosis of the disease entity (asthma), but these mechanisms may be associated with different characteristics (C1 and C2) which are then used to define the asthma phenotypes. It is also possible that the characteristics used to define the phenotypes are also used to diagnose the disease, in which case there would be arrows from C1 and C2 to Asthma. However, in the current example, we assume that the characteristics that are used to define the phenotypes (C1, C2) are not directly involved in disease diagnosis, but are only used to define the phenotypes.

In Figure S1 there are theoretically three possible phenotypes: the first is due to A1 (and identified through C1), the second is due to A2 (and identified through C2), and the third occurs when A1 and A2 coexist (if this is biologically possible).

Assuming that the simplified model of Figure S1 is correct, then the phenotypes would directly correspond to pathogenic mechanisms. The exposures may act specifically on a pathogenic mechanism (E_1_ or E_3_) or may be shared by the different pathogenic mechanisms (E_2_). It should be noted that C_1_ and C_2_ may be a set of characteristics (rather than single characteristics), but still be specific for a single pathogenic mechanism.

**Problems of causal interpretation**

***Figure S2.*** *Diagram depicting a situation in which the set of characteristics (C) used to define the asthma phenotypes are not always specifically linked to a given pathogenic mechanism (A).* ***2a)*** *E denotes an exposure potentially causing at least one of pathogenic mechanisms.* ***2b)*** *A further exposure (E_4_) causes one of the characteristics used to define the phenotypes but it is not a cause of any of the pathogenic mechanisms of interest*.


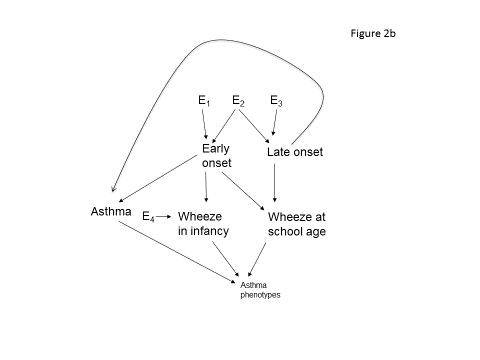

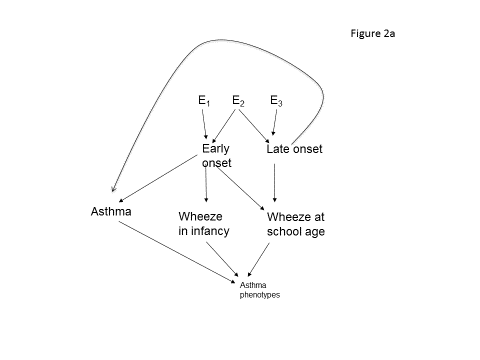


Figure S2a depicts a situation in which this assumption of direct correspondence is not met. The set of characteristics C_2_ is caused by both pathogenic mechanisms A_1_ and A_2_. This scenario, for example, could apply to asthma when phenotypes are defined on the basis of wheezing in infancy (C_1_) and/or at school age (C_2_): both the phenotype “persistent wheezing” (C_1_ and C_2_) and the phenotype “late-onset wheezing” (C_2_ only) involve the presence of C_2_. There may also be factors (e.g. E_4_ in Figure S2b) acting on some of the characteristics without acting on any of the pathogenic mechanisms of interest. Thus, in some situations the characteristics used to define the phenotype may occur also in individuals without the disease of interest. For example, there may be infections causing wheezing in infants who do not have, and will not have, asthma.

In the scenarios depicted in Figure S2, the use of the phenotypes for aetiological studies on the pathogenic mechanisms A_1_ and A_2_ can be problematic because of two possible reasons: “low specificity” and “low penetrance” [ref Richiardi et al].

***Scenario 1: Low specificity*.** Under the scenarios in Figure S2b, some individuals have the set of characteristics C_1_ for mechanisms that are unrelated with the pathogenic mechanism A_1_ (in Figure S2b, C_1_ is caused also by E_4_). If these individuals are exposed to E_3_, they can have both C_1_ and C_2_, even if they did not experience the pathogenic mechanism A_1_. Thus, the exposure E_3_ that induces only the pathogenic mechanism A_2_, may be associated also with the phenotype corresponding to the pathogenic mechanism A_1_. Using the simplified example on asthma to illustrate this problem, some of the individuals who had the *pathogenic mechanism* “late onset asthma” (A_2_) could be classified in the *phenotype* “persistent wheezing” because they had infections (E_4_) in infancy which induced infant wheezing (C_1_). Presence of this phenotype would be interpreted as presence of the *pathogenic mechanism* “persistent wheezing” (A_1_). This problem would remain the same even if: (i) E_4_ were a risk factor for a further pathogenic mechanism (A_3_) only inducing C_1_; *and* (ii) different pathogenic mechanisms could co-exist in the same diseased individual (e.g. a person could have experienced both A_2_ and A_3_).

***Scenario 2: Low penetrance****.* A further complication occurs if a pathogenic mechanism does not always produce the associated characteristics, which would weaken the correspondence between phenotypes and pathogenic mechanisms. For example, if in Figure S2a or 2b the probability of C_1_ given A_1_ were less than 100%, the pathogenic mechanism A_1_ would affect also the probability of having only C_2_. Individuals who had the *pathogenic mechanism* of persistent-asthma (A_1_) would show a *phenotype* “late-onset asthma”, if in some cases the pathogenic mechanism A_1_ did not result in wheezing in infancy.

Note that the two scenarios of low specificity and penetrance low could be considered as two aspects of the same problem: the markers C are not a perfect (or good) proxy of the pathogenic mechanisms A. In particular, lack of specificity implies that the characteristic C can occur independently of A and low penetrance means that C is not a necessary consequence of A. It is also relevant to consider that restricting on the presence or absence of a characteristic (e.g. an analysis restricted to subjects who do not experience wheezing at school age in Figure S2, i.e. C_2_=0) would not solve these two problems, as these analyses would be affected by the so-called collider bias [ref] and would thus produce spurious results.

These scenarios are hypothetical, but they indicate possible problems with the approach of Martinez et al [ref] in their 1995 paper in which they described three wheezing phenotypes: transient-early, late-onset and persistent. They found, for example, that male sex was associated with a relative risk of 1.0 (95% CI: 0.7-1.5) for early- transient wheezing, 2.1 (95% CI: 1.3-3.4) for late-onset wheezing and 1.9 (95% CI: 1.2-3.0) for persistent wheezing. This pattern would suggest that male sex affects both A_1_ and A_2_ pathogenic mechanisms in Figure S2 (i.e. male sex would be an E_2_-like risk factor). However, it can be calculated that the results similar to those of Martinez et al would be consistent, for example, with male sex affecting only the pathogenic mechanism A_2_ with a relative risk of 3.2, if 27% of the infants would experience early wheezing without having the A_1_ asthma and the probability of having wheezing at school age in children with A_1_-asthma would be 19%.

**
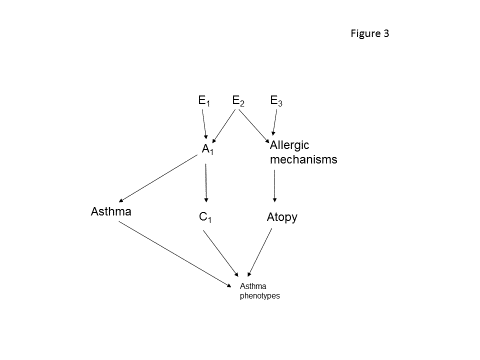
*Figure S3.*** *Diagram depicting a situation of lack of causality. The set of characteristics (C) used to define the asthma phenotypes are linked with pathogenic mechanisms (A) which are (A1) or are not (A2) causes of asthma. E denotes an exposure potentially causing at least one of pathogenic mechanisms.*

***Scenario 3: Lack of causality*.** A different problem may occur if one of the supposed pathogenic mechanisms is in fact not a cause of the disease. Figure S3 depicts such a scenario in which A_2_ is not a cause of asthma (e.g. if atopy was not a cause of asthma but merely a related condition, similar to rhinitis). The corresponding characteristic (atopy) could still have a prognostic role, but it would not be a marker of a pathogenic mechanism. Under this scenario, any risk factor for A_2_ (and thus atopy) would be associated with both disease phenotypes defined on the basis of C_2_ (presence or absence of C2) even if this risk factor does not have any aetiological role for the disease of interest.

Many would disagree with the suggestion that atopy may not be part of the causal pathway for asthma, and may just be a related condition. However, it is interesting to consider this hypothetical possibility, and to see the implications for defining phenotypes. It is well-documented that less than one-half of asthma cases are attributable to atopy, and that the other half involve non-atopic mechanisms. [ref] However, even for the cases that are associated with atopy, it is not completely clear that the association is causal; for example, atopy is only weakly associated with asthma in low-and-middle-income countries**,** which raises questions about whether the association that is seen in high income countries is causal. [ref] Figure S3 illustrates well this situation. Suppose that there was a single unifying underlying mechanism for asthma (A_1_); it is not currently clear what this is, but there may be underlying mechanisms which produce bronchospasm (C_1_) which is the key feature of an asthma attack. [ref] Other mechanisms (A_2_) could then cause atopy (C_2_) but not asthma itself. In this scenario, atopy would not be a cause of asthma; rather asthma and atopy would have common causes (E_2_). Then, classifying asthma into atopic and non-atopic phenotypes would simple produce a phenotype (atopic asthma) which was really just a marker of atopy, and would have the same risk factors as atopy. In this situation, we could expect that some factors (E3) which had no association with asthma itself, would increase the risk of atopic asthma, but decrease the risk of non-atopic asthma. In this situation (which is hypothetical, but is consistent with the findings of some studies) [refs], both the positive and negative associations would not be causal, but would be artifacts of the inappropriate classification of asthma into atopic and non-atopic phenotypes.

**Reference**

Richiardi L, Barone-Adesi F, Pearce N. Cancer subtypes in aetiological research. European Journal of Epidemiology 2017; 32: 353-361.
